# Transport and hardness properties of Cr_2_TiAlC_2_ MAX phase synthesized by reactive spark plasma sintering

**DOI:** 10.1080/14686996.2026.2675215

**Published:** 2026-07-16

**Authors:** Florent Baudouin, Cédric Bourgès, Illia Serhiienko, Théo Duchesne, Fabien Grasset, Hiroyo Segawa, Jean-François Halet, Takao Mori

**Affiliations:** a MANA, National Institute for Materials Science (NIMS), Tsukuba, Japan; b International Center for Young Scientists (ICYS), National Institute for Materials Science, Tsukuba, Japan; c CNRS, IRCER, Univ. Limoges, Limoges, France; d CNRS–Saint-Gobain–NIMS, Laboratory for Innovative Key Materials and Structures (LINK) – IRL CNRS 3629, National Institute for Materials Science (NIMS), Tsukuba, Japan; e Research Center for Electronic and Optical Materials, National Institute for Materials Science (NIMS), Tsukuba, Japan; f Univ Rennes, CNRS, Ecole Nationale Supérieure de Chimie de Rennes, Institut des Sciences Chimiques de Rennes (ISCR), Rennes, France; g Graduate School of Pure and Applied Sciences, University of Tsukuba, Tsukuba, Japan

**Keywords:** MAX phase, Cr_2_TiAlC_2_, reactive SPS, transport property, thermoelectricity, vickers hardness

## Abstract

Cr_2_TiAlC_2_ MAX phase was synthesized for the first time by reactive spark plasma sintering (SPS) from pure metallic precursors. A systematic study of each SPS parameter was performed to promote the formation of the pure phase. The transport properties of the resulting pellet were probed and compared to a conventionally synthesized sample made from pure Cr_2_TiAlC_2_ densified powder. The electrical conductivity was measured to be 8.31 × 10^5^ S.m^−1^ at 298 K, while the thermal conductivity was measured to be 11.76 W.m^−1^.K^−1^ at 298 K, one of the lowest values reported for a MAX phase. The measured Vickers hardness was 7.62 ± 0.16 GPa at a load of 19.8 N, which is about 40% higher than samples synthesized by hot pressing, making it the hardest *312* MAX phase reported to date. Although Cr_2_TiAlC_2_ was confirmed to be a poor thermoelectric compound, exhibiting a low Seebeck coefficient of −4.2 μV.K^−1^ at 298 K. These results provide a baseline for comparing structural and transport properties prior to etching into MXenes, thereby helping to better understand the influence of dimensional reduction on thermoelectric performance. Overall, these results demonstrate that reactive SPS is a rapid and efficient method for MAX phase synthesis.

## IMPACT STATEMENT

A rapid reactive SPS approach is reported, enabling high-purity, dense Cr_2_TiAlC_2_ MAX phase with refined microstructure, superior hardness, and reduced thermal conductivity compared to conventional processing routes.

## GRAPHICAL ABSTRACT

**Figure:**
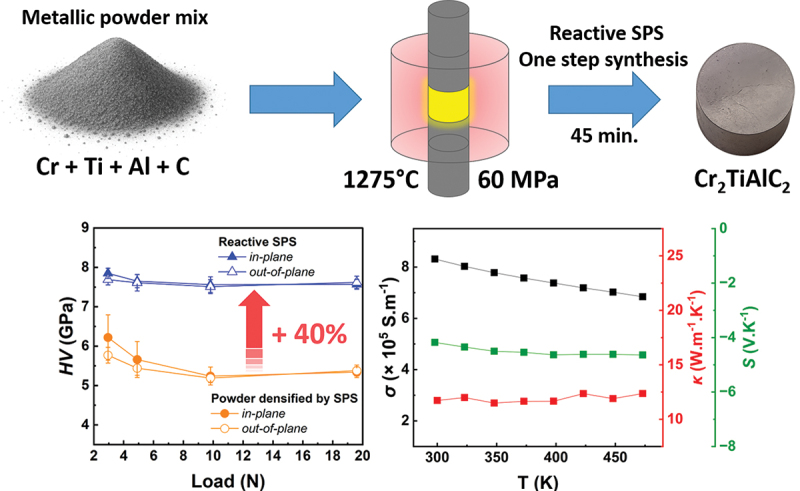

## Introduction

MAX phases are a family of layered compounds of chemical formula M_n +1_AX_n_, with *M* = transition metal, A = A-layer element (typically group 13 or 14 elements), and X = C and/or N discovered in the 1960’s [1,2]. These phases have a layered hexagonal structure, which crystallize in a *P*6_*3*_*/mmc* space group for the majority of them, composed of M_n +1_X_n_ layers separated by A-element layers. They have attracted a large research interest since they combine the characteristics of both metals and ceramics [3,4]. Among them, Cr_2_TiAlC_2_ (CTAC), discovered a decade ago, is a compound composed of abundant elements and the first discovered *o*-MAX (*out-of-plane* chemical order on the M site) [5,6]. Several studies have shown that CTAC displays good oxidative resistance (up to 1000°C under air), excellent lubricating properties and anti-wear performance, exhibits microwave absorbing properties, and especially excellent mechanical properties compared to other *312* MAX phases [7–10]. Despite its potential interest, this material remains underexplored compared to more popular MAX phases such as Ti_3_AlC_2_, Ti_3_SiC_2_, or Cr_2_AlC [11–14]. Its synthesis has indeed been limited to hot pressing (HP) and solid-state reaction [10,15], while a few of its physical properties have not yet been reported.

MAX phases are also well-known for producing 2D nanosheets, also called MXenes, through the selective etching of the A layer [16]. In particular, Cr_2_TiC_2_T_x_ MXenes obtained from the etching of CTAC remain underexplored [15,17]. Moreover, it was theoretically predicted that Cr_2_TiC_2_T_x_ MXenes could display high thermoelectric (TE) performance [18]. The thermoelectric efficiency of a material is commonly described by the dimensionless figure of merit *zT* = *S*^2^*σT*/(*κ*_latt_+*κ*_e_) where *S* is the Seebeck coefficient, *σ* the electrical conductivity, *T* the temperature, *κ*_latt_ is the lattice (phonon) contribution to the thermal conductivity, and *κ*_e_ the electronic (carrier) contribution [19,20]. Although these predictions may overestimate *zT*, they highlight the interest in investigating related parent MAX phases from an experimental perspective. Cr_2_TiAlC_2_ itself remains scarcely investigated and several of its intrinsic physical properties, including thermoelectric properties, have not yet been experimentally reported. In this context, we synthesized the Cr_2_TiAlC_2_ MAX phase and investigated its thermoelectric properties. Although the Cr_2_TiAlC_2_ MAX phase is expected to be metallic, the recent discovery of remarkably high thermoelectric power factors in some metallic systems [21–23] suggests that such materials deserve further attention.

Herein, we report the first synthesis of Cr_2_TiAlC_2_ MAX phase by reactive spark plasma sintering (R-SPS), demonstrating a rapid and efficient route to obtain this compound in high purity. By enabling simultaneous *in situ* phase formation and densification, R-SPS provides an alternative to conventional hot pressing (HP), while drastically reducing the processing time to less than 1 h and reduce the synthesis temperature. In contrast to classical SPS, which is used for the consolidation of pre-synthesized powders, R-SPS integrates synthesis and densification within a single processing step [24]. Moreover, owing to the intrinsic electrical conductivity of MAX phases, the applied current can progressively flow through the compact during reaction, thereby promoting volumetric Joule heating and enhanced reaction kinetics [25]. Despite these advantages, the application of reactive SPS to MAX phase synthesis remains relatively limited [26,27]. We systematically evaluated the physical properties of the R-SPS product, including its temperature-dependent electrical and thermal conductivities, Vickers hardness at room temperature, and microstructure. The results were compared with those of a conventionally synthesized, SPS-densified reference (D-SPS) and with literature data on samples prepared by HP. In addition, the experimental transport results of the R-SPS sample were compared to theoretical predictions by Y.F. Li et al. [28]. Pure Cr_2_TiAlC_2_ was obtained with a combination of high hardness record, high electrical conductivity, and unusually low thermal conductivity, highlighting its interest among *312* MAX phases.

## Experimental part

### Sample preparation

Cr_2_TiAlC_2_ was synthesized by mixing chromium (≥99.96%, powder, ‒200 mesh, Nacalai Tesque), titanium (99.98%, powder, <45 μm, Sigma-Aldrich), aluminum (99.99%, powder, ‒300 mesh, Rare Metallic Co.), and carbon graphite (≥99.99%, powder, <45 μm, Sigma-Aldrich) in a stoichiometric ratio 2:1:1.1:2 with a stainless-steel grinding vial containing two 10 mmØ and three 5 mmØ stainless steel balls for 3 h in a SPEX 8000D mixer/mill (Cole-Parmer, U.S.A.) under Ar atmosphere.

For the R-SPS route, the powder was directly sintered by spark plasma sintering (Dr Sinter Lab Jr. series 322 Lx, Fuji Electronic Industrial Co., LTD.) using a 10 mm diameter graphite die under a partial vacuum atmosphere. The temperature was first raised from room temperature (RT) to 873 K at a heating rate of 100 K/min as a pyrometer control step. The temperature was then raised to 1548 K at a heating rate of 75 K/min, and held for 15 min. A uniaxial pressure of 20 MPa was applied up to 873 K, followed by a gradual increase to 60 MPa (approximately 4.4 MPa/min) up to the final sintering temperature.

For the D-SPS route, the powder was placed in an alumina boat crucible and heated in a tubular furnace under Ar flow at 5 K/min at 1673 K for 5 h. The resulting powder was crushed, sieved <38 μm, treated with 9 M HCl for 2 h to remove eventual intermetallic secondary phases, and dried in an oven (≈330 K) overnight before SPS. The powder was then densified by SPS using the same sintering program as for the R-SPS sample.

All pellets were polished and cut to the required shapes and dimensions for the *in-plane* and *out-of-plane* property measurements, and the leftover pieces were crushed into fine powder (<38 μm) for powder XRD measurements.

### Characterization

The phase purity and crystal structure were characterized using a routine *θ*–2*θ* Bragg-Brentano diffractometer MiniFlex600-Cu Rigaku Corporation (Japan) with a Cu K*α*_1_ radiation source (*λ* = 1.5406Å) with a step size of 0.02° and a step time of 8°/min. For Rietveld refinement, news patterns were performed using a *θ*–2*θ* Bragg-Brentano diffractometer SmartLab3 Rigaku Corporation, Japan, with a step size of 0.01°, and a step time of 2°/min. X-ray diffraction (XRD) patterns were refined by the Rietveld refinement performed using the FullProf and WinPLOTR software packages [29,30]. The shape of the diffraction peaks was modeled using a Thompson-Cox-Hastings pseudo-Voigt profile function with axial divergence asymmetry [31,32]. Zero-point shift, asymmetry parameters, background contribution, and lattice parameters were systematically refined. As the MAX phase was characterized by a highly anisotropic morphology, the preferential orientation along the (00*l*) was considered. Finally, the fractional atomic coordinates and the isotropic displacement parameters (*i.e.*, Debye–Waller factors: B_iso_) were refined.

Observations of the fractured cross-section of the densified pellets were performed using a field emission scanning electron microscope (FE-SEM) Hitachi S-4800 (Japan). The densified pellets surfaces were observed using a high-resolution scanning electron microscope (HR-SEM) Hitachi SU8230 (Japan), equipped with an energy dispersive X-ray (EDX) detector (X-MaxN, Oxford Instrument, UK). For the latter, the surface observations and component analysis were performed on mirror-polished samples.

Thermogravimetric analysis (TGA) and differential scanning calorimetry (DSC) were carried out using a simultaneous thermogravimetric analyzer (449 F1 Jupiter, Netzsch, Germany).

The electrical conductivity (*σ*) and Seebeck coefficient (*S*) were simultaneously measured by the four-probe method from 300 to 573 K using a ZEM-3 (ULVAC Advance-Riko, Japan) device under partial helium pressure.

The temperature-dependent thermal diffusivity, *α*, and the temperature-dependent heat capacity, *C*_p_ were measured using the laser flash diffusivity method in LFA-467 Hyperflash (Netzsch, Germany) on graphite-coated square-shaped samples of 6 mm for the *in-plane* direction, and disk-shaped samples of 10 mm diameter for the *out-of-plane* direction. The total thermal conductivity was calculated using the formula, *κ* = *α* × *C*_p_ × *ρ*, where *α* is the thermal diffusivity and *ρ* is the density of the sample (measured using Archimedes method). The contributions of the electronic and lattice parts were calculated. The lattice thermal conductivity (*κ*_latt_) was estimated from *κ* by subtracting the electronic contribution (*κ*_e_) from the Wiedemann-Franz law, as in the following equation: *κ*_e_ = *L* × *σ* × *T* where *L* is the Lorenz number (Sommerfeld approximation). *κ*_latt_ was estimated by subtracting *κ* and *κ*_e_: *κ*_latt_ = *κ* – *κ*_latt_.

The carrier concentration was measured using a PPMS (Quantum Design, U.S.A.) in a magnetic field from −5 T up to +5 T at 300 K.

Vickers hardness measurements were carried out using a Shimadzu HMV-G micro hardness tester, Japan. Samples were mirror-polished with a 2000-grit abrasive paper before testing. Loads ranging from 2.9 to 19.6 N were applied, and 12 indentations were performed per load. To minimize statistical bias, the lowest and highest values were excluded, resulting in a final set of 10 measurements per load.

## Results and discussion

### Synthesis optimization

The first part of this work consisted in optimizing the sintering process using R-SPS to obtain the pure phase. For this purpose, each processing parameter was individually optimized by varying one parameter at a time while keeping the others constant. The sintering temperature range (1473–1673 K) and elemental ratios (Cr:Ti:Al:C = 2:1:1.2:1.8) were selected based on previously published studies [10,33], whereas the heating rate (50–100 K/min), sintering time (5–30 min), and applied pressure (20–60 MPa) ranges were chosen based on prior experience and established SPS practice in our laboratory. After optimizing the heating rate and sintering temperature, some leakage of metallic aluminium from the die was observed for the initial Al molar ratio of 1.2. The aluminium content was therefore adjusted to suppress this leakage, with an optimal value of 1.1. Following the optimization of the sintering time, the carbon content was also increased. However, the relatively low densities obtained at 20 MPa for the D-SPS sample led to further investigation of the effect of higher applied pressures.

The experimental optimization is summarized in the supplementary information (Table S1), and the X-ray diffraction patterns corresponding to each series of samples are also provided (Figure S1–S6). The best sample of each series was selected based on the diffractogram showing the highest purity of the MAX phase. 

**Figure 1.**
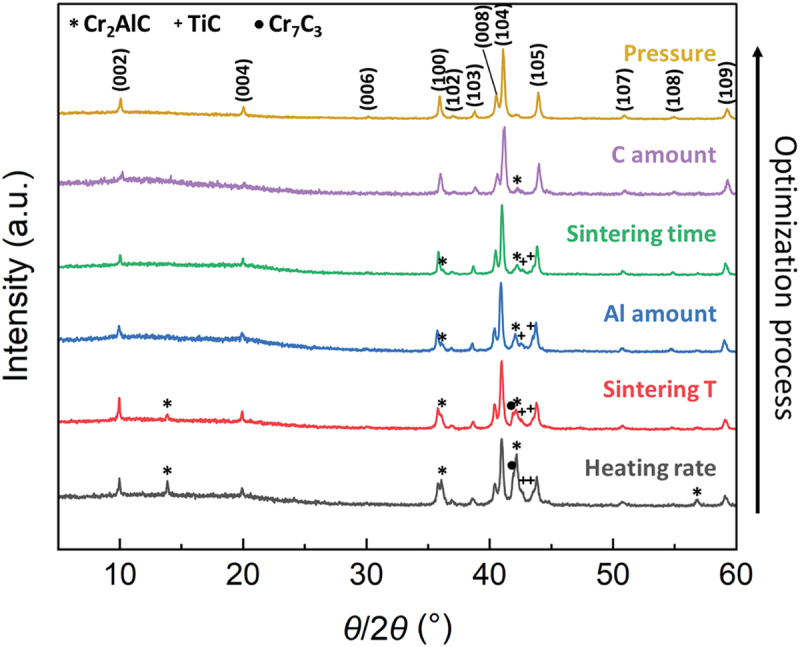
XRD patterns of the samples, during the optimization process, after SPS. In black, the sample with a heating rate optimized at 75°C/min, in red the sample with the sintering temperature optimized at 1275°C, in blue the sample with the aluminum amount optimized at a molar ratio of 1.1, in green the sample with the sintering time optimized at 15 min, in purple the sample with the carbon amount optimized at a molar ratio of 2.0, and in orange the optimized sample at 60 MPa (*i.e*., R-SPS).

Figure 1 displays the X-ray diffraction patterns of the samples from each synthesis series that showed the highest phase purity. In the initial synthesis conditions (Figure 1 - first black pattern), strong reflections from secondary phases are observed and identified as Cr_2_AlC (space group *P*6_3_/*mmc*; *a* ≈ 2.87 Å, *c* ≈ 12.86 Å), TiC (space group *Fm*$\overset{ˉ}{3}$*m*; *a* ≈ 4.60 Å), and Cr_7_C_3_ (space group *Pmcn*; *a* ≈ 7.02 Å, *b* ≈ 12.16 Å, *c* ≈ 4.53 Å). By systematically optimizing each SPS parameter independently, these undesired phases were progressively suppressed. This step-by-step optimization ultimately enabled the synthesis of the single-phase Cr_2_TiAlC_2_ MAX phase, with only minor residual peaks. The most favorable synthesis (R-SPS sample) conditions were identified as a molar ratio of Cr:Ti:Al:C = 2:1:1.1:2, a heating rate of 75 K/min, a sintering temperature of 1548 K held for 15 min, and an applied uniaxial pressure of 60 MPa. The total synthesis time is about 45 min (Figure S1 in the ESM), which is significantly lower than the 6-h synthesis in traditional HP [10,34], representing a substantial improvement in synthesis efficiency.

### Characterization of Cr_2_TiAlC_2_ R-SPS sample

**Figure 2.**
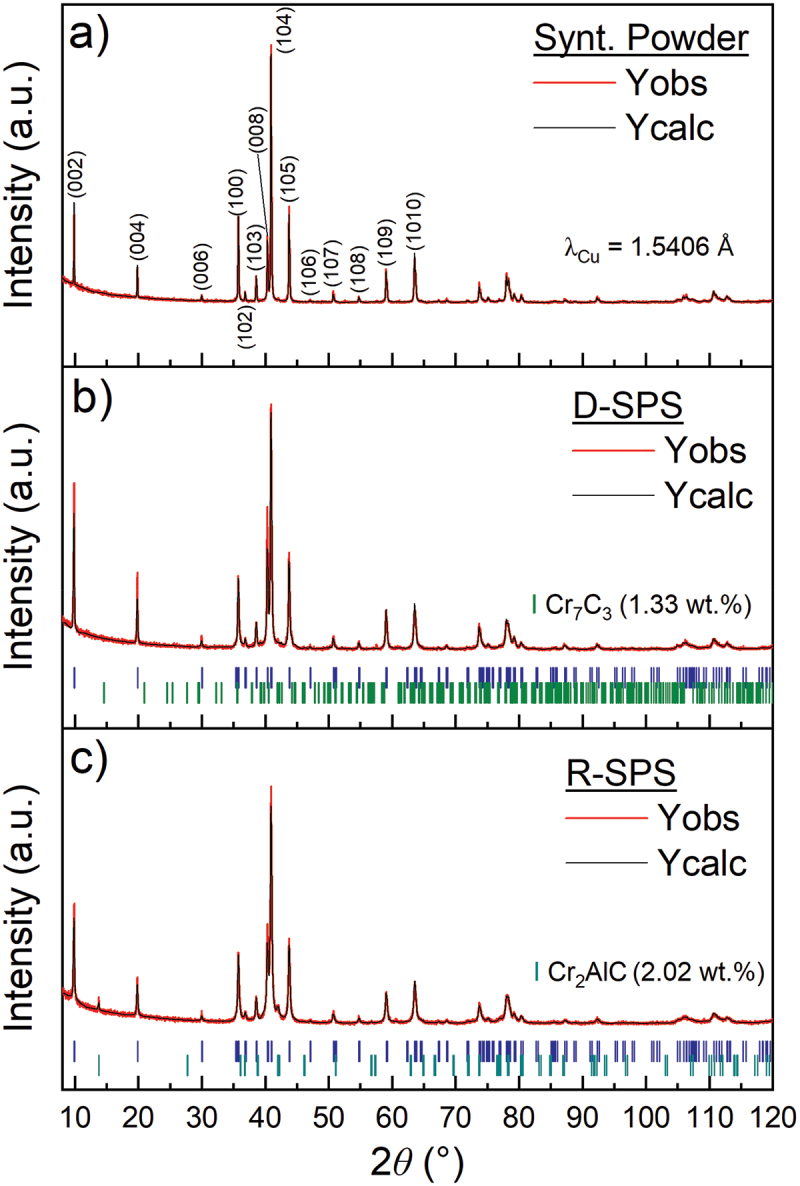
X-ray powder diffraction patterns and their corresponding Rietveld refinement fit of a) synthesized powder, b) D-SPS, and c) R-SPS sample at room temperature.

**Figure 3.**
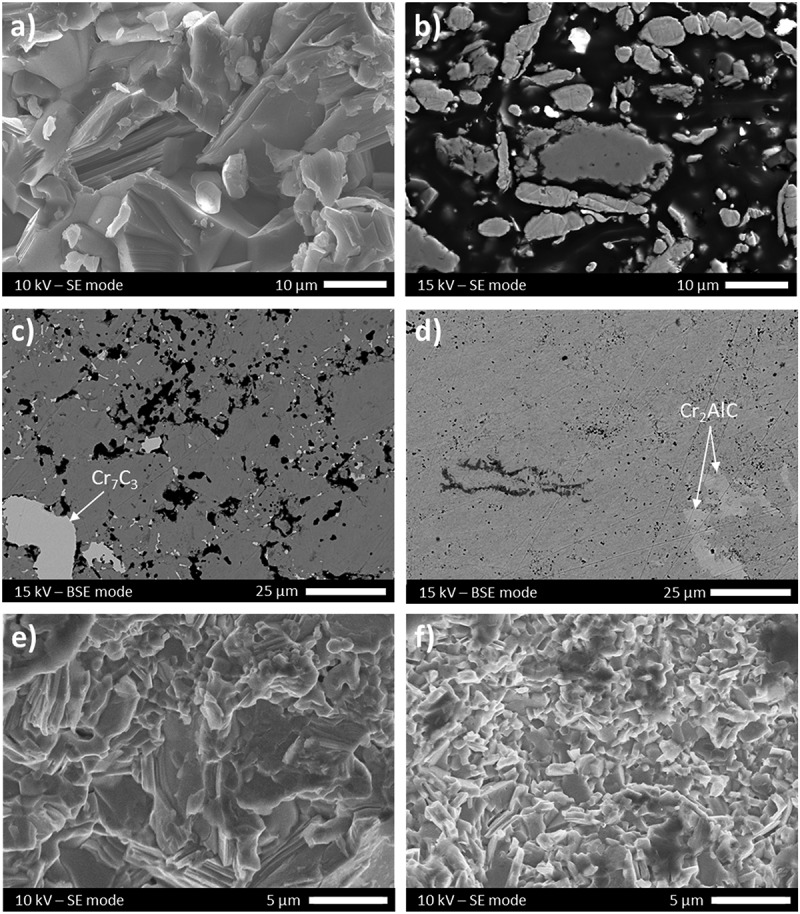
SEM micrographs of a) CTAC powder synthesized by solid state reaction, b) polished grains of the CTAC powder. c) polished D-SPS sample, d) polished R-SPS sample, e) D-SPS cross section, and f) R-SPS cross section.

The final R-SPS optimized sample was compared with a sample made of densified powder D-SPS, sintered with the same procedure (Figure S1 in the ESM). After synthesis, the measured densities of D-SPS and R-SPS pellets were 4.87 and 5.01 g.cm^−3^, respectively, with a relative density of 94.6% and 97.5% (calculated density around 5.15 g.cm^−3^). The XRD patterns and corresponding Rietveld refinement are displayed in Figure 2 and Table 1, respectively, for the synthesized powder, densified powder (D-SPS), and reactive SPS (R-SPS). Through the Rietveld refinements, the main phase in each pattern is indexed as the *312* MAX phase structure (space group *P*6_3_/*mmc*; *a* ≈2.92 Å, *c* ≈17.87 Å). The synthesized powder pattern (Figure 2a) appears exempt of secondary phases; however, a minor amount of 1.33 wt.% of Cr_7_C_3_ phase is formed after the densification by SPS (Figure 2b) and confirmed by backscattered electrons (SEM-BSE) analysis (Figure 3c). Moreover, the SPS process induced a strong increase in peak intensities at 9.9°, 19.9°, 30.0°, and 40.3° in the D-SPS corresponding to the (00*l*) indexation of the structure, indicating a slight preferential orientation of the crystallites. As the refinement considers this plan, it confirms the assumption of a plate shape morphology (*i.e*., preferential parameter <1), which is consistent with the SEM observations (Figure 3). From the crystal structure analysis point of view, it has been concluded that the Debye–Waller factors (*B*_iso_) of the synthesized powder need to be fixed for the Ti and Al site (Table S2-*B*_iso_-asterix* in the ESM) as the refinement did not converge to realistic values. This suggests that the Ti or Al atomic plans present some anomaly, such as a Ti-Al site exchange due to the ball-milling – post-annealing hybrid synthesis process. Indeed, the high-energy ball-milling step can induce some structural defects that cannot be fully relaxed by the following thermal treatment. However, the densification by SPS is a well-known reactive method, which leads to the densification and texturing of the material thanks to the high heating kinetic and uniaxial load applied on the initial compacted powder [35]. Moreover, it can also activate complex atomic diffusion mechanisms, favoring a fast crystallization, strain relaxation, or atomic reorganization. In the present case, the Rietveld refinement of the D-SPS sample converges to lower reliability factors (*R*_Bragg_ and *R*_F_) and accurate Debye-Weller factors (*B*_iso_) for Ti and Al, which attests of a fairly well *312* MAX structure arrangement sustaining our assumption (Table S3 in the ESM). In the same line, the R-SPS approach exhibited similar features to the D-SPS sample with even lower reliability factor attesting to the proficient effect of the SPS on the synthesis for promoting well-crystallized Cr_2_TiAlC_2_ MAX phase with ordered structure [6]. Moreover, we can observe an apparent lower preferential orientation than the D-SPS sample as well as the formation of 2.02 wt.% of the *211* Cr_2_AlC MAX phase in the R-SPS sample instead of Cr_7_C_3_.

**Table 1.** Refined parameters of Cr_2_TiAlC_2_ for the three synthesis methods.

Cr_2_TiAlC_2_, *P*6_3_/*mmc*, λ_Cu_							
Name	*a* (Å)	*c* (Å)	*V* (Å^3^)	χ^2^	*R*_Bragg_ (%)	*R*_F_ (%)	Secondary phases
Synt. powder	2.9259(1)	17.8669(1)	132.5(1)	1.31	7.59	6.20	–
D-SPS	2.9262(1)	17.8739(1)	132.6(1)	1.37	5.55	4.27	Cr_7_C_3_ (1.33 wt.%)
R-SPS	2.9259(1)	17.8687(1)	132.5(1)	1.04	4.20	4.03	Cr_2_AlC (2.02 wt.%)

The microstructure of the samples was examined by SEM (Figure 3) to complement the XRD analysis and assess the effect of the synthesis route on densification and grain morphology. Figure 3a shows the morphology of the CTAC powder after sieving, revealing typical layered MAX phase grains with particle sizes ranging from 5 to 10 µm. The composition of the polished powder (Figure 3b) was found to be Cr_1.9 ± 0.2_Ti_1.0 ± 0.1_Al_0.9 ± 0.1_C_2.2 ± 0.2_, which is in agreement with the theoretical stoichiometry. For the D-SPS sample (Figure 3c,e), the microstructure shows a porous structure (black areas), consistent with the lower relative density measured. A small amount of Cr_7_C_3_ secondary phase was also observed, consistent with the XRD results. The average crystallite size is about 5–10 µm, comparable to that of the starting powder. EDX analysis reveals a composition similar to that of the precursor powder, indicating that no significant change in stoichiometry occurred during densification. In contrast, the R-SPS sample exhibits a dense and homogeneous microstructure with fine crystallites (~1 µm) and minimal porosity (Figure 3d,f). Minor Cr_2_AlC second phase is present, whereas no Cr_7_C_3_ was observed, in agreement with the XRD results. EDX analysis confirms that the elemental ratios remain unchanged, indicating that the reactive SPS did not alter the stoichiometry. The R-SPS microstructure is finer and denser than that of D-SPS, reflecting the reactive synthesis process. Starting from metallic precursors, rapid elemental interdiffusion leads to fine crystallization and a compact MAX-phase structure. These microstructural characteristics are expected to affect the material’s physical properties, as will be discussed in the following sections.

**Figure 4.**
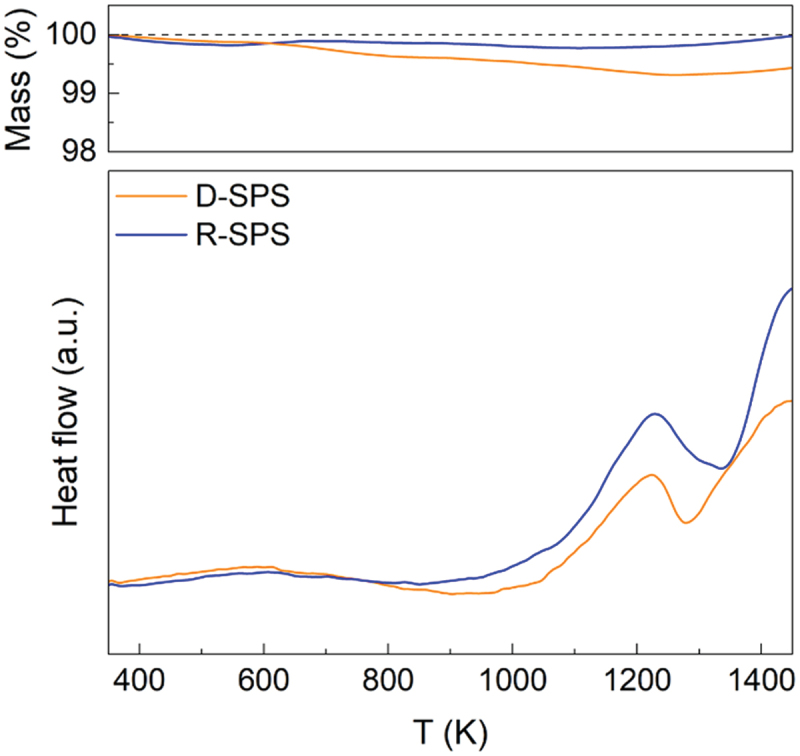
Differential scanning calorimetry (DSC) and thermogravimetric analysis (TGA) results for D-SPS and R-SPS samples.

The thermal stability of the samples was investigated by DSC and TGA, as shown in Figure 4. For both samples, the observed weight loss is below 1%, which is within the instrumental uncertainty range. In the DSC heat flow curves, both D-SPS and R-SPS exhibit an endothermic peak corresponding to the formation of secondary phases, in agreement with previous reports [36]. However, the peak temperature is slightly higher for R-SPS (~1230 K) than for D-SPS (~1220 K), indicating a slightly enhanced thermal stability of the R-SPS sample. The onset temperature, defined as the intersection between the baseline and the tangent to the leading edge of the peak, also differs: ~1140 K for D-SPS and ~950 K for R-SPS. This suggests that, while the phase transformation occurs in the same general temperature range, its initiation is influenced by sample-specific factors. This includes (i) the R-SPS synthetic route, which can produce metastable microstructures with residual stresses that can lower the local activation barrier [37]; (ii) the presence of minor Cr_2_AlC phase in R-SPS, which can promote the dissociation of Cr_2_TiAlC_2_ into Cr_2_AlC + TiC phases that are thermodynamically favored below 1350 K [10]; and (iii) the finer crystallite size in R-SPS (1 µm vs 5 µm for D-SPS) which increases the grain-boundary area and enhances solid-state diffusion and nucleation rates [38]. Overall, the very low mass loss (<1%) observed in TGA confirms that both samples remain thermally stable up to 1200 K.

### Transport properties

**Figure 5.**
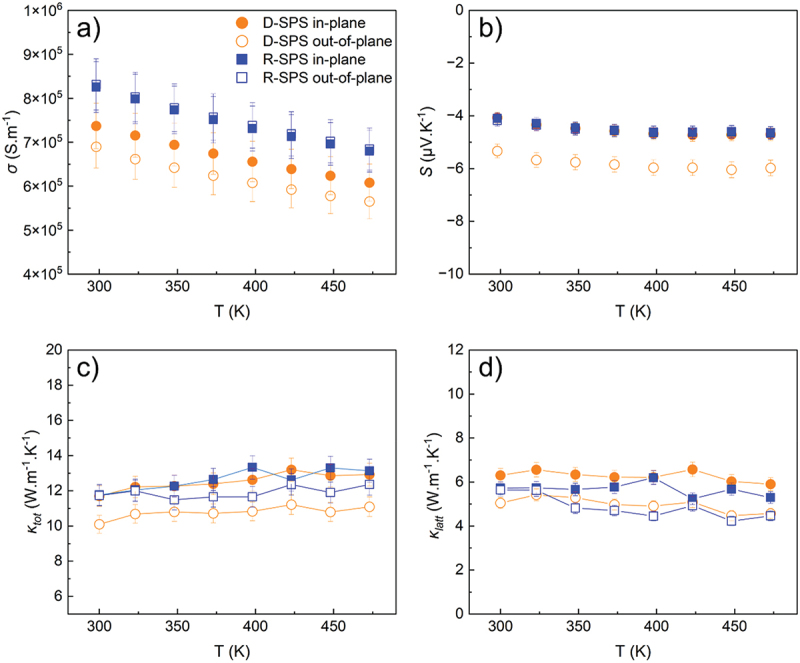
a) Electrical conductivity of the samples as a function of the temperature. b) Seebeck coefficient as a function of the temperature. c) thermal conductivity of both samples as a function of the temperature. d) lattice thermal conductivity of both samples as a function of the temperature. Both samples were measured in the *in-plane* (full dots) and *out-of-plane* (empty dots) directions. In blue is the R-SPS sample, and in orange is the D-SPS sample.

The *in-plane* and *out-of-plane* electrical conductivities (*σ*) were measured to assess the possible anisotropy of charge transport in Cr_2_TiAlC_2_, which may arise from its layered crystal structure and the texturing induced during densification (Figure 5a). *In-plane* measurements were performed perpendicular to the SPS pressing axis (within the pellet plane), whereas o*ut-of-plane* measurements were carried out parallel to the pressing axis. Considering the lamellar structure of the MAX phase and the uniaxial nature of SPS processing, any anisotropy in the transport properties would be associated with a possible texture induced along the pressing direction. The D-SPS sample shows a slight anisotropy, with *in-plane* and *out-of-plane* conductivities of 7.4 × 10^5^ and 6.9 × 10^5^ S.m^−1^ at 298 K, respectively, corresponding to a 7% difference. Such anisotropy comes from the slight alignment of larger crystallites during the SPS process, as determined by XRD refinement, which enhances directional transport along the basal planes. In contrast, the R-SPS sample displays nearly isotropic electrical behavior, with both *in-plane* and *out-of-plane* conductivities reaching 8.31 × 10^5^ S.m^−1^ at 298 K. The absence of anisotropy is consistent with its finer and more randomly oriented microstructure, as confirmed in SEM and XRD analyses. The difference in electrical conductivity between the two samples can be mainly attributed to their respective densities (4.87 g.cm^−3^ for D-SPS and 5.01 g.cm^−3^ for R-SPS); the lower density of D-SPS naturally results in lower conductivity. In addition, minor secondary phases may also slightly contribute to this difference, as Cr_7_C_3_ exhibits a significantly lower electrical conductivity than Cr_2_AlC [39]. As summarized in Table 2, the measured conductivities are compared with the previously reported data in the literature at room temperature. To our knowledge, the electrical conductivity of Cr_2_TiAlC_2_ has only been reported for samples synthesized by hot pressing, with values ranging from 6.5 to 8.4 × 10^5^ S.m^−1^ [10,34]. The conductivity measured for the R-SPS sample lies near the upper limit of this range, indicating that reactive SPS can achieve performance comparable to the best hot-pressed samples. All these experimental values exceed the calculated electrical conductivities in both directions [28]. Compared with other *312* MAX phases, CTAC exhibits relatively low electrical conductivity, slightly higher than that of Mo_2_TiAlC_2_ (4.8‒7.7 × 10^5^ S.m^−1^ at 298 K) [54,55].

**Table 2.** Summary of the experimental data reported for Cr_2_TiAlC_2_ and other *312* max phases at 298 K. Cr_2_AlC and Mo_2_Ti_2_AlC_3_ were added for their close composition and for their remarkable properties, respectively.

MAX phases	*σ* (×10^6^ S.m^−1^)	*S* (V.K^−1^)	*κ* (W.m^−1^.K^−1^)	*HV* (GPa)	References
Cr_2_AlC	1.4	1‒3.3	17.9‒22	3.5‒5.2	[14,40–42]
Ti_3_AlC_2_	3.5‒3.6	5	40	2.7‒4.1	[11,43–48]
Ti_3_SiC_2_	4.4‒4.5	0.18‒2	37–43	3‒5.8	[12,45,49–53]
Ti_3_GeC_2_	4.5	2	39	2.2‒5	[13,49,51]
Mo_2_TiAlC_2_	0.48‒0.77	‒	8	5.5‒7.5	[54–56]
Cr_2_TiAlC_2_ (calc.)	0.21 (*out-of plane*) 0.57 (*in-plane*)	‒11	0.5 (*out-of-plane*) 18.7 (*in-plane*)	‒	[28]
Cr_2_TiAlC_2_ (HP)	0.65‒0.84	‒	14.9	5‒5.6	[10,34]
Cr_2_TiAlC_2_ (D-SPS)	0.69 (*out-of-plane*) 0.74 (*in-plane*)	‒5.3 (*out-of-plane*) ‒4.1 (*in-plane*)	10.1 (*out-of-plane*) 11.7 (*in-plane*)	5.4	This work
Cr_2_TiAlC_2_ (R-SPS)	0.83	‒4.1	11.7	7.6	This work
Mo_2_Ti_2_AlC_3_	0.41‒0.42	‒15	6.8	4.8	[45,57]

The Seebeck coefficient of each sample was measured simultaneously with the electrical conductivity (Figure 1b). It reflects the material’s ability to convert a temperature gradient into an electrical voltage providing insight into its potential thermoelectric performance. For the R-SPS sample, no difference was observed between the *in-plane* and *out-of-plane* directions, consistent with the previous structural and electrical results. The Seebeck coefficient remained relatively low, ranging from −4.1 microV.K-1 at RT to −4.7 μV.K^−1^ at 423 K. On the other hand, the D-SPS sample exhibited anisotropy in the Seebeck coefficient, consistent with the slight anisotropy observed in the XRD refinement and electrical conductivity. The *in-plane* Seebeck response of D-SPS closely matched that of the R-SPS sample, while *the out-of-plane* values ranged from −5.3 μV.K^−1^ at RT to −6.1 μV.K^−1^ at 448 K. Although these values fall below the theoretical prediction (−11 μV.K^−1^) proposed for polycrystalline Cr_2_TiAlC_2_ [28], they remain within the range typically reported for MAX phases, as summarized in Table 2. These low Seebeck coefficients can be explained by the electronic band structure of MAX phases. DFT calculations realized on Ti_3_AlC_2_, Ti_3_SiC_2_, and Ti_3_GeC_2_ indicate that Ti 3*d*-derived states near the Fermi level (*E*_*f*_) hybridize with C 2*p* and A *np* orbitals (*n* = 3 for Al and Si, *n* = 4 for Ge), producing anisotropic dispersions: flatter bands along the *c*-axis and more dispersive bands in the basal plane, leading to *in-plane* and *out-of-plane* Seebeck coefficients with opposite signs, nearly canceling in polycrystalline samples [58–60]. Cr_2_TiAlC_2_ is also expected to display an anisotropic Seebeck, as the DFT calculations show similar anisotropic bands near the Fermi level, with 3*d*–Cr states hybridized with 3*d*–Ti, 3*p*–Al, and 2*p*–C orbitals [33]. Notably, the measured Seebeck coefficient of Cr_2_TiAlC_2_ remains negative over the temperature range studied, indicating a near compensation between electron and hole contributions, with a slight predominance of the electron contributions. Furthermore, the carrier concentration of both CTAC samples was measured at room temperature. Similar values were obtained: 3.54 × 10^21^ cm^−3^ for the D-SPS sample and 3.58 × 10^21^ cm^−3^ for the R-SPS sample, consistent with a metallic electronic structure. As a result, the Seebeck coefficient is intrinsically low, in agreement with the expected behavior of such systems.

The thermal conductivity was evaluated as a function of temperature for both Cr_2_TiAlC_2_ samples (Figure 5c) to further correlate heat transport with the previously discussed structural and electronic characteristics. A weak anisotropy is observed for the D-SPS sample, with higher *in-plane* than *out-of-plane* conductivity (10.1 W.m^−1^.K^−1^ vs 11.7 W.m^−1^.K^−1^ at RT), consistent with the texturing identified by XRD and the electrical anisotropy previously discussed. In contrast, the R-SPS sample exhibits nearly isotropic heat transport, in line with its randomly oriented, fine-grained microstructure, as confirmed by SEM and XRD. Both SPS-synthesized samples exhibit lower *κ* than the values reported for hot-pressed Cr_2_TiAlC_2_ (14.9 W.m^−1^.K^−1^ at 298 K [34]), which is primarily attributed to their smaller grain size and increased phonon scattering at grain boundaries. Li *et al*. theoretically predicted that the *in-plane* and *out-of-plane* thermal conductivities of Cr_2_TiAlC_2_ are *κ*_a_ = 18.7 W.m^−1^.K^−1^ and *κ*_c_ = 0.5 W.m^−1^.K^−1^, respectively [28], for a perfectly oriented single crystal. These values differ significantly from the experimental values, reflecting the strong influence of real-microstructure effects on heat transport, such as incomplete texturing, grain boundaries, and residual porosity. The electronic contribution (*κ*_e_) was calculated from the Wiedemann-Franz law and the phonon contribution *κ*_latt_ from the difference between *κ*_tot_ and *κ*_e_. The results of *κ*_latt_, reported in Figure 5d, show that it represents around 50% of the *κ*_tot_, which is consistent with the results obtained on a HP sample (~58%) [34], demonstrating that phonons and electrons equally contribute to heat transport. The thermal conductivity of Cr_2_TiAlC_2_ is compared with other MAX phases reported in the literature (Table 2). Compared to other *312* MAX phases, Cr_2_TiAlC_2_ exhibits one of the lowest thermal conductivities, exceeded only by Mo_2_TiAlC_2_ (8 W m^−1^.K^−1^ at RT), while the lowest value reported for any MAX phase is that of Mo_2_Ti_2_AlC_3_ (6.8 W.m^−1^.K^−1^ at RT) [61].

### Mechanical properties

**Figure 6.**
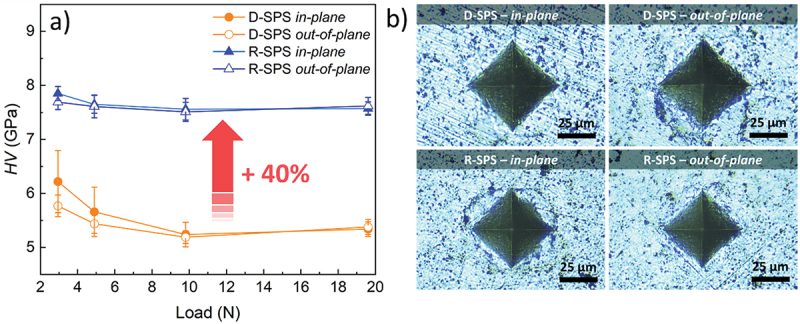
Vickers hardness as a function of the load applied to the Cr_2_TiAlC_2_ samples. Both samples were measured in the *in-plane* (full dots) and *out-of-plane* (empty dots) directions. In blue is the R-SPS sample, and in orange is the D-SPS sample. b) Vickers indentation impressions on the polished surface of each sample.

Finally, the Vickers hardness (*HV*) of D-SPS and R-SPS samples (Figure 6) was measured to compare with reported values for CTAC synthesized by hot HP [10,34], and to assess the potential impact of microstructural differences on their mechanical properties. For the D-SPS sample, the hardness decreased from 6.22 to 5.34 GPa for the *in-plane* sample and from 5.77 to 5.38 GPa for the *out-of-plane* sample, as the load increased from 2.94 to 19.61 N, in agreement with the indentation size effect reported in the literature [10,62,63]. This effect, characterized by higher hardness values and greater data dispersion at low loads, is related to grain size, dislocation density, and elastic – plastic properties [64]. At a load of 19.61 N, the D-SPS samples display values of 5.34 ± 0.14 GPa for the *in-plane* orientation and 5.38 ± 0.14 GPa for the *out-of-plane* orientation, which is close to the values reported elsewhere for Cr_2_TiAlC_2_ [10,34]. However, for the R-SPS sample, this effect is slightly observed for the *in-plane* direction, while it is not observed in the *out-of-plane* sample. At a 19.61 N load, the Vickers hardness was measured to be about 7.57 ± 0.12 GPa (IP) and 7.62 ± 0.16 GPa (OP), representing an improvement of about 40% compared to the D-SPS sample and the literature. The difference in density between the samples does not appear to play a key role in this hardness enhancement, since the *HV* values are similar for the HP-synthesized sample reported in the literature and D-SPS-synthesized samples (5.02 and 4.87 g·cm^−3^, respectively) [10]. This significant increase in hardness observed in the R-SPS sample can be attributed to a smaller grain size compared to the D-SPS sample, as shown previously on the SEM micrographs (Figure 2). For the D-SPS sample, the grain size is about 5 µm, while it is about 1 μm for R-SPS. Reducing grain size increases the number of grain boundaries, which act as obstacles to dislocation motion inside the material. This phenomenon, described by the Hall–Petch relationship, implies that both mechanical strength and hardness increase as grain size decreases:

$$\sigma_{y}=\sigma_{0}+\frac{k_{y}}{\surd d}$$

where *σ*_y_ is the yield stress, *σ*_0_ represents the intrinsic resistance of the lattice to dislocation motion, *k*_y_ is the strengthening coefficient, and *d* is the average grain size.

The Vickers indentation impressions on the polished surfaces of each sample (Figure 6b) further illustrate the differences in hardness. The D-SPS samples show slightly larger indentation marks than the R-SPS samples, consistent with their lower hardness. Minor radial cracks are occasionally observed around the indentations. Such features are common in hard materials exhibiting localized cracking under stress and do not affect the hardness measurement [65]. Overall, the indentations are well-defined, confirming the reliability of the *HV* data.

The Vickers hardness of CTAC is compared to other MAX phases in Table 2. Most of the MAX phases display a hardness between 4 and 5 GPa. In our case, the R-SPS sample displays the highest Vickers hardness among the *312* MAX phases. However, it is important to note that very close values were obtained for the Mo_2_TiAlC_2_ phase (7.5 GPa), and a similar grain size was observed for the measured sample (1–3 µm) [56].

## Conclusion

In this study, we have demonstrated for the first time that the *312* Cr_2_TiAlC_2_ MAX phase can be efficiently synthesized *via* reactive SPS (R-SPS) in a very short processing time (≈45 min). The resulting R-SPS samples exhibit the highest purity reported so far, a dense microstructure with grain sizes around 1 µm, and no preferential crystallite orientation. These structural features translate into an enhanced Vickers hardness of 7.6 GPa, which is about 40% higher than that obtained by hot pressing, making it the hardest reported *312* MAX phase. This exceptionally high hardness confirms the potential of Cr_2_TiAlC_2_ for applications in protective coatings, wear-resistant components, and structural materials designed for high-temperature or harsh environments. The electrical conductivity of the R-SPS sample reaches 8.3 × 10^5^ S.m^−1^, while the thermal conductivity is 11.8 W.m^−1^.K^−1^ at room temperature, one of the lowest in the *312* MAX phase family. The Seebeck coefficient remains low with ‒4.2 μV.K^−1^ at room temperature, indicating poor thermoelectric performance in the bulk MAX phase, but providing a baseline for comparison with Cr_2_TiC_2_T_x_ MXenes, which may exhibit enhanced thermoelectric properties upon dimensional reduction. Overall, the results described here highlight that reactive SPS is a rapid and effective method for easy MAX phases synthesis with improved mechanical and transport properties.

## Supplementary Material

Supplemental Material
