# Supplementary material for: Transport and hardness properties of Cr2TiAlC2 MAX phase synthesized by reactive spark plasma sintering
Source: Sci Technol Adv Mater. 2026 Jul 16;27(1):2675215. doi: 10.1080/14686996.2026.2675215 (PMC13378725; doi:10.1080/14686996.2026.2675215)
Supplement: Supplemental Material [file TSTA_A_2675215_SM3305.docx]

**ELECTRONIC SUPPLEMENTARY MATERIAL**

**Transport and hardness properties of Cr_2_TiAlC_2_ MAX phase synthesized by reactive spark plasma sintering**

Florent Baudouin^1^, Cédric Bourgès^3,4^, Illia Serhiienko^1^, Théo Duchesne^1,2^, Fabien Grasset^2^, Hiroyo Segawa^2,5^, Jean-François Halet^6^, Takao Mori^1,7,^*

*^1^ National Institute for Materials Science (NIMS), MANA, 1-1 Namiki, Tsukuba, 305-0044, Japan ^2^ CNRS–Saint-Gobain–NIMS, IRL 3629, Laboratory for Innovative Key Materials and Structures (LINK), National Institute for Materials Science (NIMS), 1-1 Namiki, Tsukuba, 305-0044, Japan*

*^3^ International Center for Young Scientists (ICYS), National Institute for Materials Science, 1-1 Namiki, Tsukuba, 305-0044, Japan*

*^4^ Univ. Limoges, CNRS, IRCER, UMR 7315, F-87000 Limoges, France*

*^5^ Research Center for Electronic and Optical Materials, National Institute for Materials Science (NIMS), 1-1 Namiki, Tsukuba, 305-0044, Japan*

*^6^ Univ Rennes, CNRS, Ecole Nationale Supérieure de Chimie de Rennes, Institut des Sciences Chimiques de Rennes (ISCR), UMR6226, F–35000, Rennes, France*

*^7^ Graduate School of Pure and Applied Sciences, University of Tsukuba, 305–8671, Tsukuba, Japan*

*Corresponding author. E-mail: [MORI.Takao@nims.go.jp](mailto:MORI.Takao@nims.go.jp)

Table 1. Summary of best purity sample of each series. The fixed parameter of each series is underlined.

| **Sample** | **Cr** | **Ti** | **Al** | **C** | **Heating rate (K/min)** | **Sint. temp. (K)** | **Sint. time (min)** | **Pressure (MPa)** |
| --- | --- | --- | --- | --- | --- | --- | --- | --- |
| Black | 2 | 1 | 1.2 | 1.8 | **75** | 1250 | 10 | 20 |
| Red | 2 | 1 | 1.2 | 1.8 | 75 | **1275** | 10 | 20 |
| Blue | 2 | 1 | **1.1** | 1.8 | 75 | 1275 | 10 | 20 |
| Green | 2 | 1 | 1.1 | 1.8 | 75 | 1275 | **15** | 20 |
| Purple | 2 | 1 | 1.1 | **2** | 75 | 1275 | 15 | 20 |
| Orange | 2 | 1 | 1.1 | 2 | 75 | 1275 | 15 | **60** |


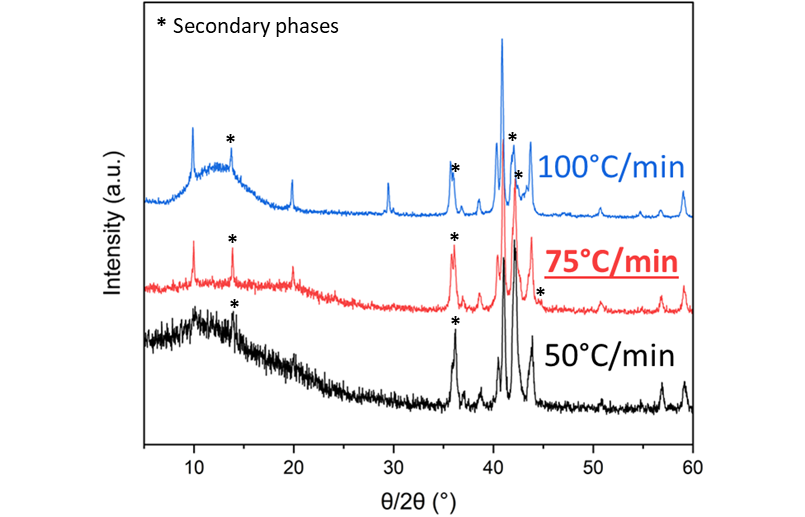


Figure S1. XRD patterns of the sample series prepared at different heating rates. All samples were sintered at 1250°C with a dwell time of 10 min under a uniaxial pressure of 20 MPa, and had a Cr:Ti:Al:C molar ratio of 2:1:1.2:1.8, as listed in Table S1.


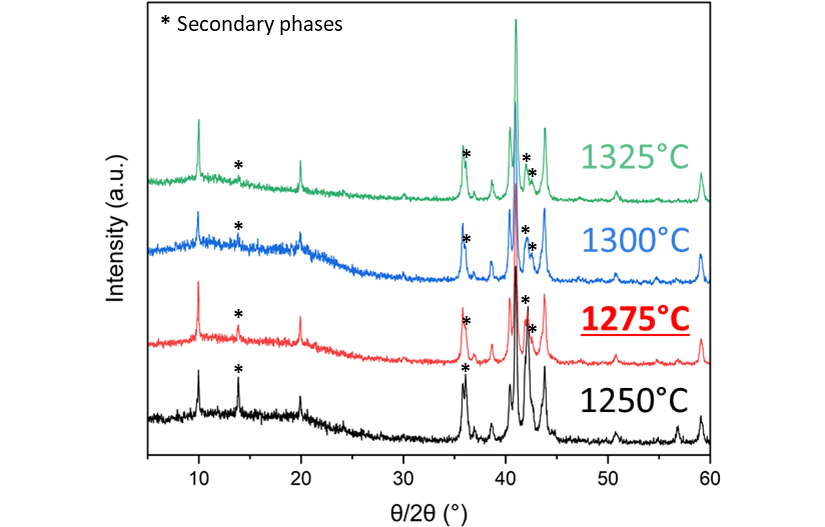


Figure S2. XRD patterns of the sample series prepared at different sintering temperatures. All samples were sintered at a heating rate of 75°C/min to the target temperature with a dwell time of 10 min under a uniaxial pressure of 20 MPa, and had a Cr:Ti:Al:C molar ratio of 2:1:1.2:1.8, as listed in Table S1.


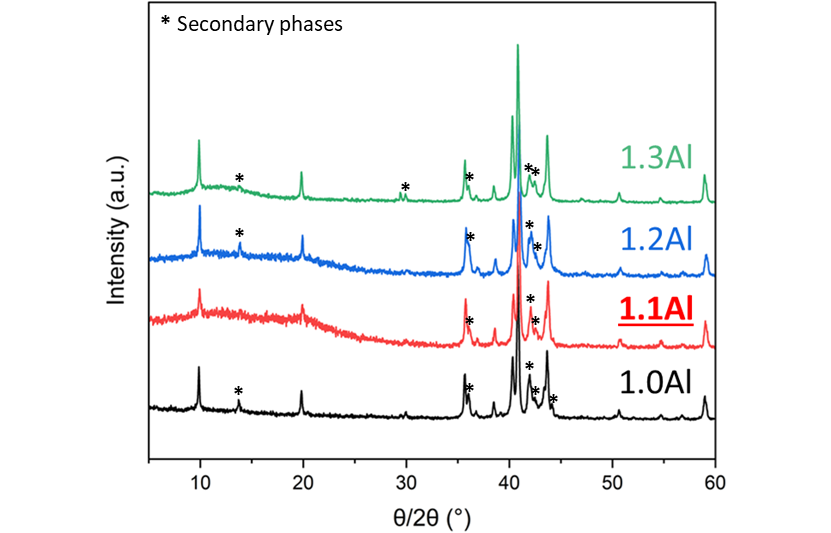


Figure S3. XRD patterns of the sample series prepared with varying aluminium contents. All samples were sintered at a heating rate of 75°C/min up to 1275°C with a dwell time of 10 min under a uniaxial pressure of 20 MPa, and had a Cr:Ti:Al:C molar ratio of 2:1:x:1.8, as listed in Table S1.


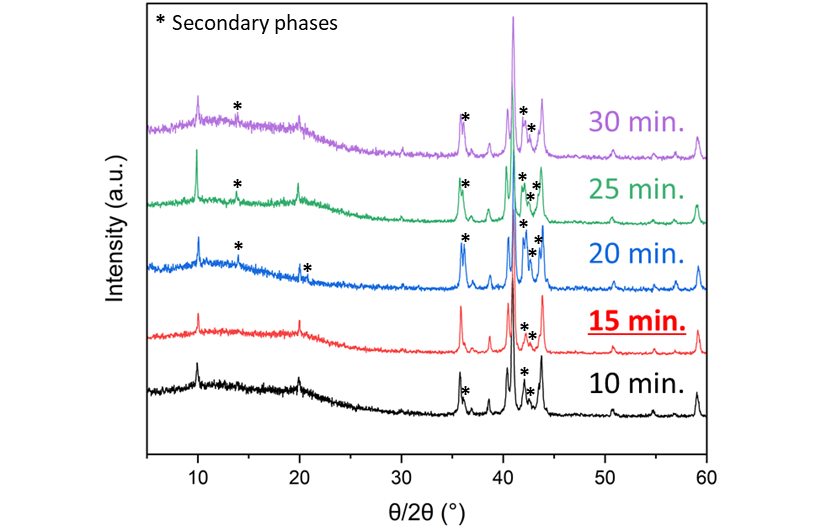


Figure S4. XRD patterns of the sample series prepared with varying sintering dwell times. All samples were heated at a rate of 75°C/min up to 1275°C and held for the specified dwell time under a uniaxial pressure of 20 MPa, with a Cr:Ti:Al:C molar ratio of 2:1:1.1:1.8, as listed in Table S1.


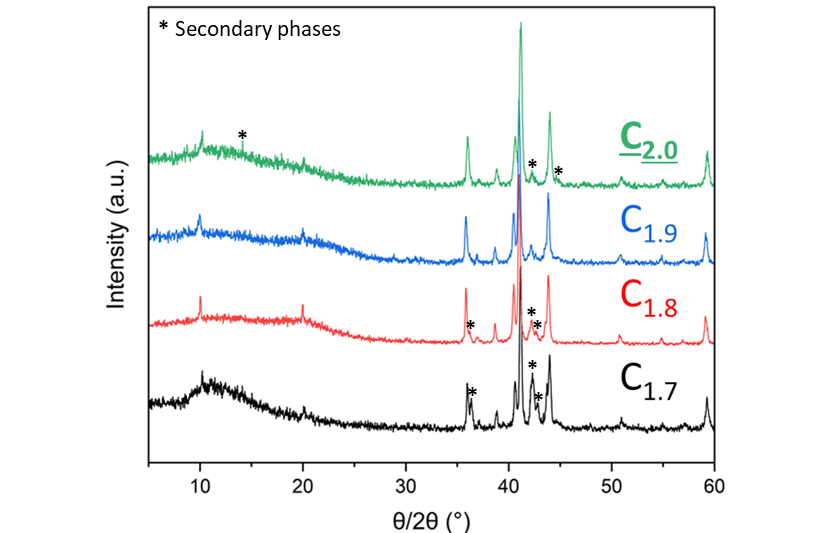


Figure S5. XRD patterns of the sample series prepared with varying carbon contents. All samples were sintered at a heating rate of 75°C/min up to 1275°C with a dwell time of 15 min under a uniaxial pressure of 20 MPa, and had a Cr:Ti:Al:C molar ratio of 2:1:1.1:x, as listed in Table S1.


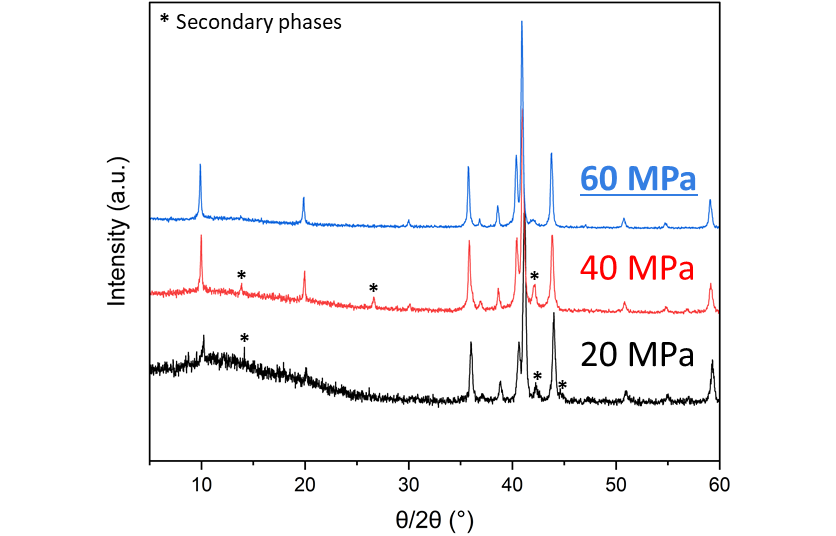


Figure S6. XRD patterns of the final sample series prepared at different sintering pressures. All samples were sintered at a heating rate of 75°C/min up to 1275°C with a dwell time of 15 min, and had a Cr:Ti:Al:C molar ratio of 2:1:1.2:2, as listed in Table S1.


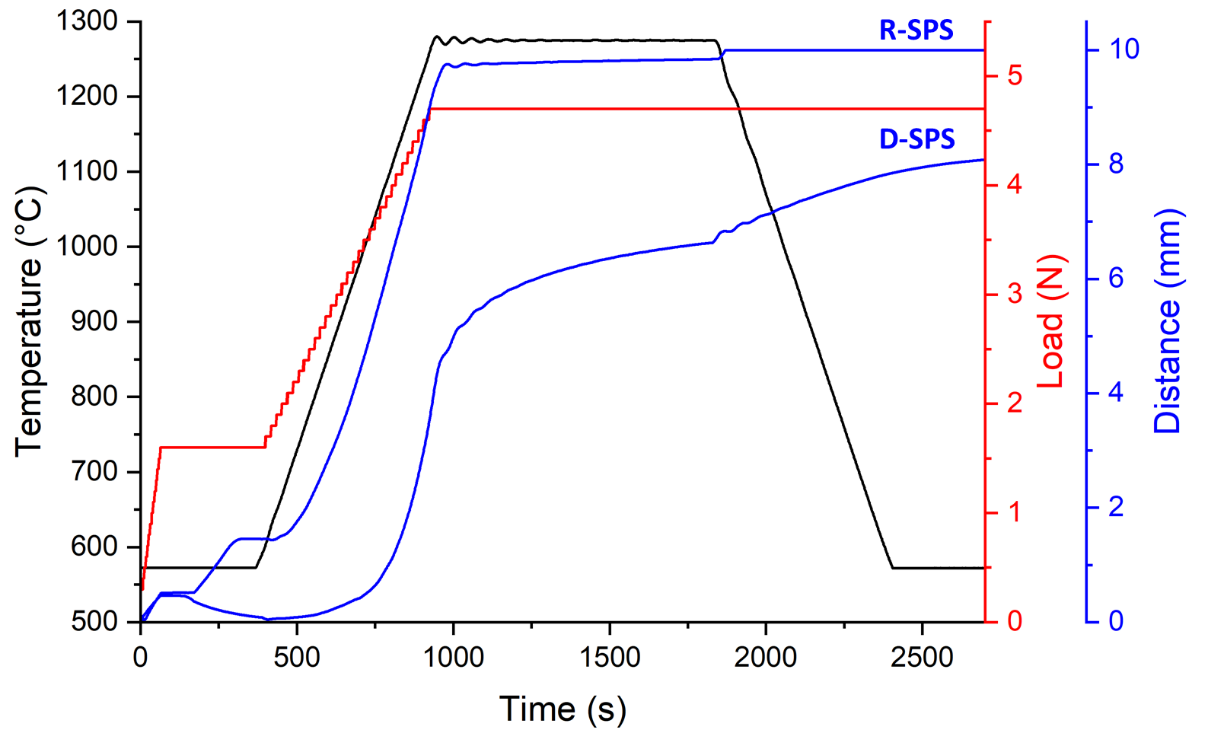


Figure S7. Samples’ behavior and sintering profile during the SPS process. The black curve represents the programmed temperature, the red curve the applied load on the powder compact, and the blue curve the punch displacement (each sample is labeled on the graph).

Table S2. Crystal structure analysis of Cr_2_TiAlC_2_ powder.

| **Synthesized Powder** | | | | | |
| --- | --- | --- | --- | --- | --- |
| ***Atom*** | ***x*** | ***y*** | ***z*** | ***Biso*** | ***Occ.*** |
| Cr | 1/3 | 2/3 | 0.1325 | 0.167 | 1 |
| Ti | 0 | 0 | 0 | *0.100 | 1 |
| Al | 0 | 0 | 0.2500 | *0.100 | 1 |
| C | 1/3 | 2/3 | 0.5772 | 0.194 | 1 |

Table S3. Crystal structure analysis of Cr_2_TiAlC_2_ powder densified by SPS (D-SPS).

| **D-SPS** | | | | | |
| --- | --- | --- | --- | --- | --- |
| ***Atom*** | ***x*** | ***y*** | ***z*** | ***Biso*** | ***Occ.*** |
| Cr | 1/3 | 2/3 | 0.1319 | 0.317 | 1 |
| Ti | 0 | 0 | 0 | 0.189 | 1 |
| Al | 0 | 0 | 0.2500 | 0.074 | 1 |
| C | 1/3 | 2/3 | 0.5775 | 0.451 | 1 |

Table S4. Crystal structure analysis of Cr_2_TiAlC_2_ synthesized by reactive SPS (R-SPS).

| **R-SPS** | | | | | |
| --- | --- | --- | --- | --- | --- |
| ***Atom*** | ***x*** | ***y*** | ***z*** | ***Biso*** | ***Occ.*** |
| Cr | 1/3 | 2/3 | 0.1317 | 0.270 | 1 |
| Ti | 0 | 0 | 0 | 0.269 | 1 |
| Al | 0 | 0 | 0.2500 | 0.059 | 1 |
| C | 1/3 | 2/3 | 0.5770 | 0.461 | 1 |

Table S5. EDX atomic compositions (at.%) and the corresponding calculated stoichiometry of Cr_2_TiAlC_2_ samples (powder, D-SPS, and R-SPS).

| **Sample** | **Cr** | **Ti** | **Al** | **C** |
| --- | --- | --- | --- | --- |
| Powder | 31.6 % | 16.1 % | 15.6 % | 36.7 % |
| Calc. stoichiometry | 1.90 | 0.97 | 0.93 | 2.20 |
| D-SPS | 30.9 % | 15.9 % | 15.3 % | 37.9 % |
| Calc. stoichiometry | 1.86 | 0.95 | 0.92 | 2.27 |
| R-SPS | 31.9 % | 16.3 % | 16.1 % | 35.7 % |
| Calc. stoichiometry | 1.91 | 0.98 | 0.97 | 2.14 |
